# Radiobiological Studies of Microvascular Damage through In Vitro Models: A Methodological Perspective

**DOI:** 10.3390/cancers13051182

**Published:** 2021-03-09

**Authors:** Luca Possenti, Laura Mecchi, Andrea Rossoni, Veronica Sangalli, Simone Bersini, Alessandro Cicchetti, Maria Laura Costantino, Christian Candrian, Chiara Arrigoni, Tiziana Rancati, Matteo Moretti

**Affiliations:** 1 Prostate Cancer Program, Fondazione IRCCS Istituto Nazionale dei Tumori, 20133 Milan, Italy; alessandro.cicchetti@istitutotumori.mi.it (A.C.); tiziana.rancati@istitutotumori.mi.it (T.R.); 2 Regenerative Medicine Technologies Laboratory, Servizio di Ortopedia e Traumatologia, Ente Ospedaliero Cantonale, 6900 Lugano, Switzerland; laura.mecchi@mail.polimi.it (L.M.); andrea.rossoni@mail.polimi.it (A.R.); veronica.sangalli@mail.polimi.it (V.S.); simone.bersini@eoc.ch (S.B.); christian.candrian@eoc.ch (C.C.); chiara.arrigoni@eoc.ch (C.A.); 3 Department of Chemistry, Materials and Chemical Engineering “Giulio Natta”, Politecnico di Milano, 20133 Milan, Italy; marialaura.costantino@polimi.it; 4 Facoltà di Scienze Biomediche, Università della Svizzera Italiana, 6900 Lugano, Switzerland; 5 IRCCS Istituto Ortopedico Galeazzi, Cell and Tissue Engineering Laboratory, 20161 Milan, Italy

**Keywords:** radiotherapy, in-vitro model, microvasculature, microenvironment, organ-on-chip, ionizing radiation, radiobiological models

## Abstract

**Simple Summary:**

Ionizing radiation is used as a treatment for cancer, but it also affects the endothelial cells that make up the microvasculature. In-vitro models can be used to study the detrimental effect of irradiation on those cells. This systematic review analyzed the literature models, highlighting the critical components of the production, irradiation, and analysis of radiobiological in-vitro models for microvascular endothelial cell damage. Based on those data, we suggest future directions, including advanced in-vitro models to recapitulate microenvironment features. We pinpoint essential information to be included for the good characterization of the experiments, especially in terms of the dose delivered by ionizing radiation.

**Abstract:**

Ionizing radiation (IR) is used in radiotherapy as a treatment to destroy cancer. Such treatment also affects other tissues, resulting in the so-called normal tissue complications. Endothelial cells (ECs) composing the microvasculature have essential roles in the microenvironment’s homeostasis (ME). Thus, detrimental effects induced by irradiation on ECs can influence both the tumor and healthy tissue. In-vitro models can be advantageous to study these phenomena. In this systematic review, we analyzed in-vitro models of ECs subjected to IR. We highlighted the critical issues involved in the production, irradiation, and analysis of such radiobiological in-vitro models to study microvascular endothelial cells damage. For each step, we analyzed common methodologies and critical points required to obtain a reliable model. We identified the generation of a 3D environment for model production and the inclusion of heterogeneous cell populations for a reliable ME recapitulation. Additionally, we highlighted how essential information on the irradiation scheme, crucial to correlate better observed in vitro effects to the clinical scenario, are often neglected in the analyzed studies, limiting the translation of achieved results.

## 1. Introduction

Ionizing radiation (IR) is widely used as a treatment for cancer. Even if treatments are optimized to minimize off-target delivered IR, non-tumor tissues suffer IR damage, likely resulting in radiation toxicity [1,2]. The IR damage to endothelial cells (ECs) composing the microvasculature is particularly troublesome since ECs are present both in tumor and healthy tissues, playing an essential role in shaping the microenvironment (ME). This damage is even more critical considering the pivotal role of ME and its complexity in determining treatment outcome, which has been recently identified [3].

Several models can be used to study the IR-damage to ECs. In this review, we focus on in-vitro models. Such models involve the replication of a biological function outside a living organism (e.g., animals). Cells are their crucial component. They are usually isolated (either in-house or commercially) and then grown in a biological laboratory [4]. Importantly, these models allow the use of cells from human sources, avoiding possible inter-species differences related to animal models. Conversely, standard models may fail in the complete recapitulation of biological phenomena such as structure, cell population, and systemic effects, among others. For this reason, in-vitro models evolved in the last decade towards more complex setups to recapitulate important features, leading to 3D culture systems [5] and organ-on-chip technologies [6,7,8,9].

This systematic review focuses on critically analyzing in-vitro models for radiobiological studies of IR-related damage to ECs composing the microvasculature. We are interested in defining such models’ key components, highlighting their importance and their use in the literature. Manuscripts were selected by systematic searches (see material and methods section). The research activity was motivated by the central role of microvasculature and ME when considering radiotherapy treatments.

The usual paradigm of in-vitro modeling is composed of model production, the application of a treatment or a stimulus, and the analysis of the sample (see, for instance, [6,7]). Given the focus of the study, irradiation (at least) always characterized the treatment. Therefore, the review is organized following the three fundamental steps required for a successful radiobiological in-vitro model [10]: (i) model production, (ii) irradiation, and (iii) analysis.

## 2. Production of the Models

In this initial step, researchers must define their in-vitro model’s basic features: the EC source and type, how to mimic the ME, and the model’s geometry.

### 2.1. EC Source and Type

**Figure 1:**
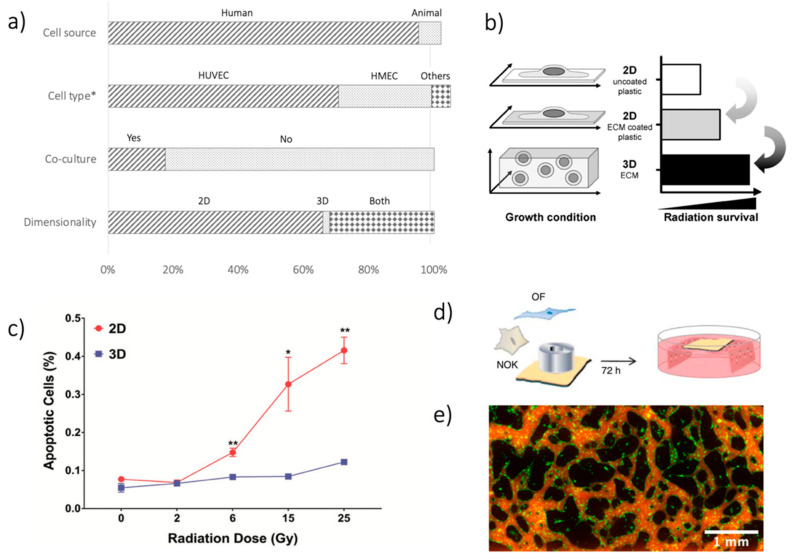
(**a**) Summary of analysis in terms of cell source (human vs. animal); cell type (HUVECs, HMECs, others); co-culture systems (yes vs. no); dimensionality of the models (2D, 3D, both). The First 2 bars exceed 100%, given that in some studies, multiple options were considered, e.g., using various cell types. *Cell type analysis is limited to human sources. (**b**) Difference in IR-related damage when considering 2D and 3D culture systems—reprinted from [22], with permission from Elsevier. (**c**) Results from [23], showing different apoptosis on EC culture after IR, as a function of the delivered dose (Gy), when using 2D and 3D cultures. Reprinted with permission from [23], © 2019 WILEY-VCH Verlag GmbH & Co. KGaA, Weinheim, Germany. (**d**) Schematic of the TEOM in-vitro model used in [24]. Reprinted under a Creative Commons Attribution-Non-Commercial-Share Alike 3.0 Unported License. (**e**) Images of GFP-positive EC (green) organized in a microvascular network on a chip and its perfusion by fluorescent dextran (red), from unpublished data of the authors. * *p* < 0.05, ** *p* < 0.01.

The EC source is the very first critical choice to be made. One hundred and forty out of 147 (95.2%) papers considered in this review used ECs from human sources (Figure 1a). ECs from animals have been reported in ten studies (6.8%). Only three studies (2.0%) made use of both EC sources. Such a choice reflects one of the main advantages of the in-vitro modeling approach, i.e., avoiding possible human-animal differences.

When considering human sources, human umbilical vein ECs (HUVECs) were the most used cells (Figure 1a), being considered in 99 out of 140 studies (70.7%). HUVECs are particularly suited for in-vitro modeling due to their easy isolation from the umbilical vein and their robustness, easy culture, and high proliferation rate [11,12]. On the other hand, HUVECs do not originate from a microvascular bed. To overcome this limitation, Human Microvascular ECs (HMECs) can be isolated from different vascular districts (e.g., dermal [13,14], brain [15,16], cardiac [17], lung [18]), even if they were less used, being present in 40 out of 140 papers (28.6%). Finally, in eight works (5.7%), different kinds of EC were used, such as EC progenitors [19] and Human Coronary EC [20].

Up to now, HUVECs and HMECs represented the two most diffused choices for EC, preferring HUVECs for cell management and availability and HMECs for recapitulating district-specific microvascular features. Even though no study directly reported a HUVEC-HMEC comparison, some similarities and some differences emerged from the few studies involving both of them, including a different activation of adhesion molecules. In particular, following IR application, ICAM-1 was upregulated on both HUVEC and HMEC, whereas E-selectin expression increased only in dermal HMEC [21].

### 2.2. Microenvironment Recapitulation

The ME can be recapitulated in-vitro by using different approaches, including the addition of other cell types, namely a co-culture, and the dimensionality of the culture, i.e., 2D or 3D cultures.

Regarding cell composition, EC monoculture is easier to perform and analyze (no need to distinguish cell types during analyses), and no influence from other cell types is present. The high number (121/147, 82.3%) of monocultures in the papers considered reflected these advantages (Figure 1a). The use of co-cultures to improve ME recapitulation was still limited, being considered in only 26 articles (17.7%). The use of a co-culture was driven by different aims, from enhancing the recapitulation of the ME [25], spanning to the ability to perform specific tests [26], to the inclusion of bystander effects of radiation (see next section), or EC-cancer cell interactions [27]. When analyzing the cell types involved in co-cultures, most studies used cancer cells (12/26, 46.2%). Possible other options were white blood cells (5/26, 19.2%), stem cells (5/26, 19.2%), and different cell types to recapitulate a specific ME, such as fibroblast or astrocytes (4/26, 15.4%).

The second option for a better microenvironment recapitulation involves model dimensionality, whereby traditional 2D culture (e.g., flask, wells) is easier to perform and analyze. 2D techniques were used in 144 out of 147 studies (98.0%). However, differences in cell organization occur when culturing them on a planar substrate as compared to a more physiological 3D environment, which is particularly important when considering the effects of IR-induced damage [22,23,28]. Indeed, cells in 2D culture suffer from significantly greater IR-induced damage than cells in 3D culture (Figure 1b,c). Notwithstanding, 3D techniques to irradiate ECs in-vitro are still rare. Most of the studies involving a 3D culture technique did not embed ECs in a 3D environment during irradiation, but only during the IR effects analysis (see angiogenesis tests in Section 4). Recently, Guo and colleagues proposed a validation paper for a 3D platform to treat microvascular networks with IR in vitro, confirming the different effects of a 3D vs. 2D environment on apoptosis, inflammation, and DNA damage induced by IR [23].

### 2.3. Non-Biological Part

To support cell culture in vitro, traditionally flasks or wells are considered [14,17,29,30,31]. The firsts are used typically for 2D culture techniques. Conversely, small wells enable the implementation of a 3D culture, given their smaller volume and the possibility to fill them with hydrogels serving as a scaffold for cell culture.

A peculiar case of 3D culture exploits de-cellularized tissues as scaffolds, in which ECs and other cell populations can be seeded and cultured, such in the case of a tissue-engineered oral mucosa model for evaluating the effects of IR induced damage [32] (Figure 1d). The Transwell technology represents another easy option to generate a co-culture. Such a device (mainly for 2D culture) creates two different environments, separated by a semi-permeable membrane. Based on the type of separation, these devices can be used for various purposes, such as the culture of two different cell types in the two compartments or permeability and migration analyses on cell seeded on the membrane (see Section 4) [33,34,35,36,37,38,39].

More advanced models are also available, in which the non-biological part of the model is specifically designed. Particularly for ECs, such models are getting more popular in the last decade, taking advantage of microfluidic technologies and supporting 3D cultures. These devices allow the production of perfusable networks on a chip, opening new possibilities in ME recapitulation and available tests (Figure 1e). However, few devices are currently commercially available as devices ready-to-use. Therefore, researchers usually produce them in-house by using polydimethylsiloxane (PDMS) via soft-lithography, stitching them to a coverslip glass by plasma treating. In these cases, the model geometry can be designed according to the particular experiment’s specific requirements. We refer readers interested in these methods to [11,40]. Although several research groups worldwide now use these models, we found only one example of their application to IR damage evaluation, nevertheless evidencing their advantages and potentiality in this field [23].

## 3. Irradiation

Once the in-vitro models are produced, they can be irradiated with IR. In this section, we analyze the studies in terms of (i) the IR source, (ii) the IR energy and irradiation phantoms, (iii) the dose delivered and the dose rate, (iv) the direct irradiation vs. the bystander effect (i.e., damage evaluated in cells directly targeted with IR vs. damage in cells out of the field of irradiation), and (v) the possible fractionation of the dose (i.e., IR is delivered on multiple days, named multiple fractions, to allow repair of sub-lethal damage within two fractions).

### 3.1. IR Sources

Once the in-vitro models are produced, they can be irradiated with IR. In this section, we analyze the studies in terms of crucial IR setup parameters.

**Figure 2:**
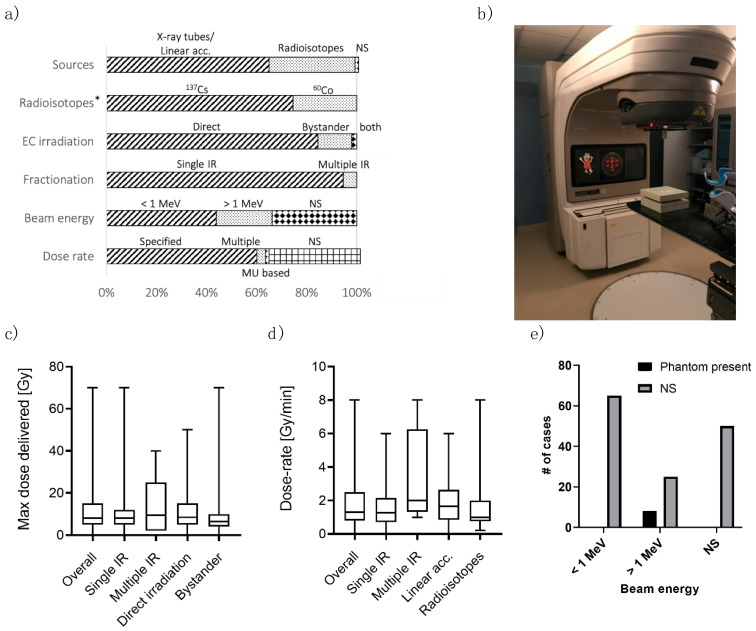
(**a**) Summary of the analysis in terms of (i) IR sources; (ii) Radioisotopes; (iii) modality of EC irradiation; (iv) possible fractionation of irradiation; (v) energy of the photon beam; and (vi) dose rate. * 100% is 51 studies, which involve radioisotope as IR source. (**b**) Picture of a DBX Varian linear accelerator, with an IR phantom placed to irradiate the sample. (**c**) Boxplot showing the distribution of the maximum dose delivered [Gy] in the studies. Max dose is also analyzed as a fractionation function (Single IR/ Multiple IR) and irradiation type (Direct/ Bystander). (**d**) Dose rate distribution in the studies. Dose rate is also analyzed as a fractionation function (Single IR vs. Multiple IR) and the source of IR (X-ray tubes/Linear accelerator vs. Radioisotopes). (**e**) Presence of the IR phantom related to the beam’s energy (less than 1 MeV, higher than 1 MeV, and not specified-NS).

Photon beams are usually generated either using X-ray tubes/linear accelerators (LINACs, Figure 2b) or from radioisotopes. In the formers, electrons are accelerated towards a target, generating photons when hitting it. The photon beam presents a spread in its energy, with maximum energy related to the electron acceleration. In radioisotopes, photons are released by an unstable nucleus while transitioning towards a stable nuclear configuration. This process produces photons of one/two selected energies. When considering the clinical practice, Mega-voltage linear accelerators are the current standard to deliver photon radiotherapy, with some centers still using cobalt-60 teletherapy [41].

In this analysis, X-ray tubes/LINACs represented the most common IR source, used in 95 out of 147 studies (64.6%, Figure 2a). Radioisotopes were employed in 51 works (34.7%). Only one study [42] has considered both types of sources to compare photon beams of different energies (see next paragraph), also considering ion irradiation.

Two radioisotopes were found in the studies: cesium-137 (^137^Cs) and cobalt-60 (^60^Co). 38 out of 51 studies (74.5%) reported ^137^Cs, which emits 662 keV photons when decaying. ^60^Co radiates 1.17 and 1.33 MeV photons (13/51 studies, 25.5%).

### 3.2. Beam Energy and Phantoms

From X-ray spectrum to γ radiation (i.e., from less energetic to more energetic photons), the energy of the photon beam determines its interaction with (biological) materials [43]. The most common forms of interaction through which photons release energy are the photoelectric effect, Compton scattering, and pair production. The probability of these events depends on the absorbing medium and the photon energy. The photoelectric effect is dominant for low energy photons (<100 keV), and its probability increases dramatically with the atomic number (Z) of the material. The Compton effect predominates for moderate to high energy photons (>100 keV). Consequently, different beams may result in various damages on the ECs. For this reason, photon energy represents a fundamental variable to be reported when describing experiments. However, 50 out of 95 (52.6%) studies using LINACs did not specify the photon energy (Figure 2a), arising difficulties in comparing results from different papers. Of note, this information’s inclusion is not required when the IR source is a radioisotope (which emits photons of characteristic energy).

The build-up process is a second important phenomenon that depends on photon energy. When interacting with materials, photons transfer energy to electrons through a first “one-shot” interaction in which a significant part of the photon energy is transferred. The electrons acquire sufficient energy to escape the atomic bond and travel through the material. While traveling, they lose their energy in a series of interactions, which transfers a relatively small amount of energy.

When describing the amount of energy released in a material as a function of depth (i.e., the distance from the surface where the photon beam is pointed), two regions can be distinguished (which arise from the two-step interaction previously described). There is an initial region where the absorbed dose increases with depth, called the build-up region. This region’s extension increases with increasing photon energy and is almost negligible for photons in the keV range. The build-up region is characterized by an electronic disequilibrium condition, making the computation of dose less accurate. After the build-up, electronic equilibrium is reached, and the absorbed dose decreases with depth following exponential attenuation. The sample should be placed downstream of the build-up region to have an accurate estimate of the dose. To this aim, a build-up phantom is needed. In particular, such a phantom (Figure 2e) is required when dealing with high-energy beams. Conversely, with low-energy beams, e.g., 200 keV, the build-up region is reduced to <2 mm [44], and the non-biological portion of the in-vitro model is enough to reach the electronic equilibrium at the EC depth. Only eight studies (5.4%) reported the presence of an irradiation phantom. All these cases ([23,25,45,46,47,48,49,50]) used beams with energy greater than 1 MeV. In all the other cases (94.6%), the phantom was not mentioned in the paper, possibly leading to an inaccurate dose estimation, especially when using high-energy photon beams.

### 3.3. Dose and Dose Rate

The dose delivered to the samples is a fundamental parameter when performing irradiation, and hence it was specified in all the studies. It is computed through different methods, from correction-based to model-based algorithms and Monte Carlo simulations [51]. Our review does not cover this topic, but we refer interested readers to [52].

The median value of the maximum dose delivered in the studies (Figure 2c) was 8 Gy, with an interquartile range of 5–13.5 Gy and ranging from a few cGy up to 70 Gy. Such values should be compared to the usual dose found in clinical practice, which may differ between tissues since the dose distribution in a radiotherapy treatment is not uniform and between different applications. We also analyzed the maximum dose delivered considering single irradiation vs. multiple irradiations. The two distributions were similar, and the median values were 8 Gy for both single irradiation and multiple irradiations. The interquartile ranges were also comparable, 5–12 Gy and 2–15 Gy, respectively. The same considerations also apply comparing direct irradiation (median 9 Gy, interquartile: 5–15 Gy) vs. bystander effect (median 6 Gy, interquartile 4–10 Gy). Overall, this range of dose values was consistent with the clinical applications (possibly also considering fractionation–see the last paragraph of this section). However, the dose(s) appropriateness should be evaluated considering the clinical scenario the in-vitro model wants to reproduce.

As opposed to the delivered dose amount, only 95 studies (64.6%) reported the dose rate. More in detail, five papers (3.4%) used multiple-dose rates, 88 (59.9%) reported a single dose rate, and two (1.4%) specified the dose rate by using the LINAC monitor units (Figure 2a). The median dose rate used in the studies was 1.3 Gy/min (Figure 2d), the total range and the interquartile ranges were 1.4 mGy/h–8 Gy/min and 0.8–2.3 Gy/min, respectively. The distribution of dose rates seems slightly different when considering single irradiation versus multiple irradiations (median 1.3 and 2 Gy/min, respectively). However, such a difference is not statistically significant (Mann-Whitney test for independent test, *p*-value = 0.09). Of note, only five studies were found involving multiple irradiations and reporting the dose rate. The IR source classification showed similar results: the median dose rate was 1.65 Gy/min for X-ray tubes/LINACs and 1 Gy/min for radioisotopes (*p*-value = 0.35, Figure 2d). Ebrahimian and colleagues [53] showed the dose rate’s effect on HUVEC functions and inflammatory response. With a higher dose rate, an increase in cytokine production, such as IL-6, MCP-1 and TNF-a, and lower in-vitro vasculogenesis capacity, was found. However, their study involved very low dose-rates to simulate chronic irradiation (from 1.4 to 4.1 mGy/h). To our knowledge, the extension of their conclusions to higher dose rates is still to be verified.

Lastly, we point out that treatments considering very high dose rates (>40 Gy/s) are emerging. Such techniques, known as FLASH, were not considered by any of the papers selected for this review, but we refer interested readers to [54,55,56].

### 3.4. Direct Irradiation and Fractionation

Irradiation can be performed directly to ECs, referred to as direct irradiation, or on other cells then put in contact with ECs. In the latter case, the study’s focus is often the bystander effect, i.e., possible IR-induced biological effects in cells not directly irradiated. Most of the works involved direct irradiation (131/147, 89.1%). Thirteen studies (8.8%) considered the bystander effect originating mainly from cancer cells [34,35,57,58,59,60,61,62,63,64], with few cases involving other cell types, such as ECs [65], blood mononuclear cells [66], and EC progenitors [19]. Different methods can be used to achieve contact between the two components or cell lines, such as direct co-culture or conditioned medium from irradiated cells to culture ECs, carrying substances produced by the other cell type following irradiation. When studying IR’s indirect effect, researchers should keep in mind that in-vitro models cannot recapitulate systemic effects, limiting the study of indirect effects on cells or components included in the model. On the other hand, such systems allow selecting the phenomena to be studied, differently from what can be obtained in vivo, where this control is hardly obtained.

Another possible difference in irradiation modality is the fractionation scheme. Most of the studies (141/147, 95.9%) involved single irradiation (Figure 2a). Anyway, even if this represents the simplest irradiation scheme to be applied, it is rarely used clinically. Clinical practice is usually characterized by fractionated radiotherapy, i.e., the total dose is split into multiple treatments called fractions given over multiple days (and even weeks). This modality allows time for normal cells to repair sublethal damage, thereby reducing side effects. Only six studies involved fractionation (4.1%). These works were heterogeneous in terms of the total dose, the number of fractions delivered, and time between doses, not allowing a direct comparison between them (three and six fractions in 3 days [67], 4 × 4 Gy [68], 5 × 2Gy [31,69], 10 × 2 Gy [70], and 20 × 1 Gy [71]). In this context, Lee and colleagues [49] compared single and fractionated irradiation using in vivo and in-vitro models, showing lower damage to the vascular system in vivo with fractionated schemes. However, they did not perform fractionated irradiation in-vitro. To our knowledge, the effect of fractionation on ECs has still to be appropriately studied.

## 4. Analyzing Results

**Figure 3:**
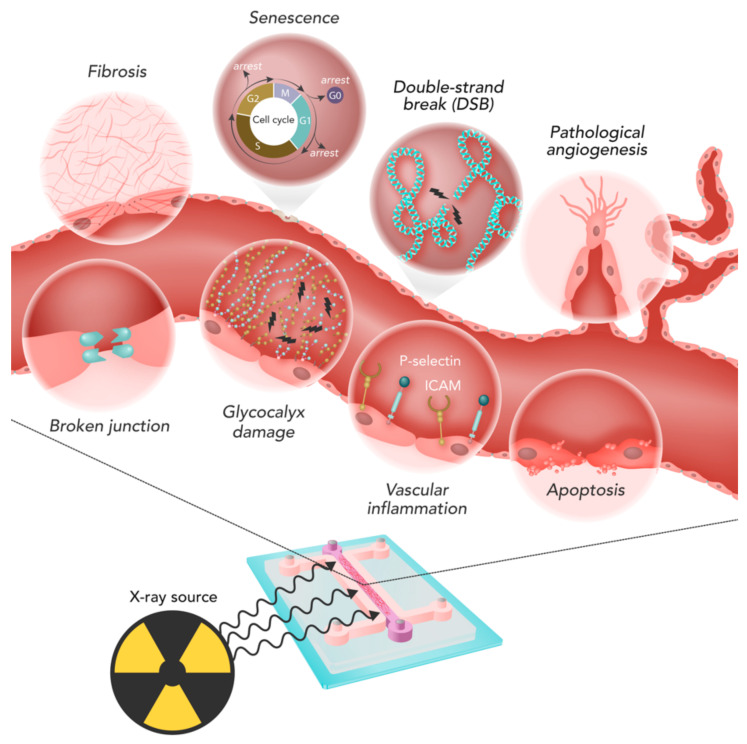
Main phenomena involved in ECs damage due to irradiation: apoptosis, pathological angiogenesis, inflammation, DNA damage, in particular double-strand breaks (DSB), glycocalyx damage, senescence, broken endothelial junctions, and fibrosis.

Once the in-vitro model is produced and irradiated, it must be analyzed to study the induced damage. Figure 3 summarizes the main phenomena involved in IR-induced ECs damage. In the following paragraphs, the different kinds of analyses, their outcome, and commonly-used techniques are discussed and described.

### 4.1. Cell Response Assays

**Figure 4:**
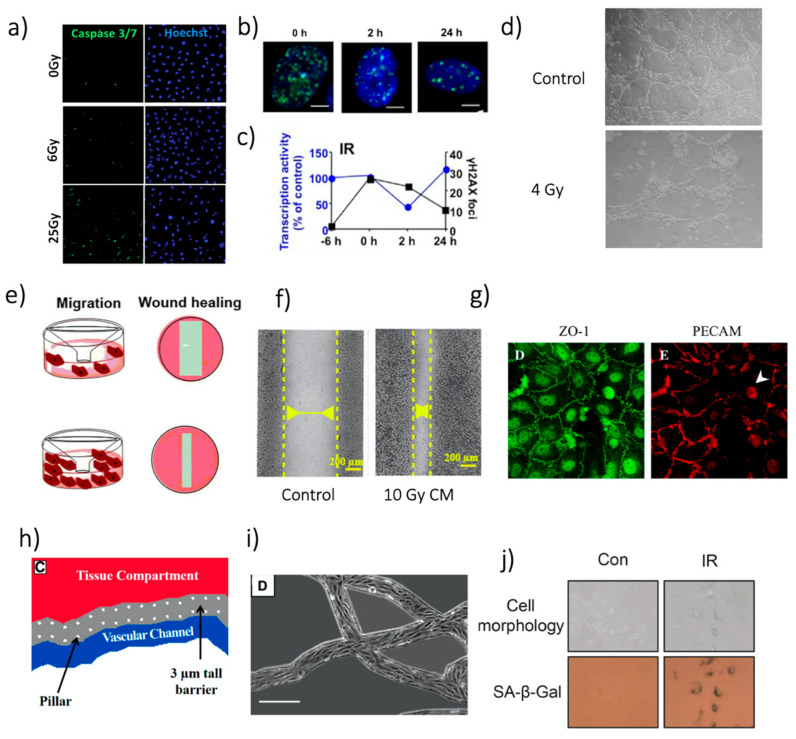
Representative images of different analyses. (**a**) Images of HUVEC apoptosis staining by caspase 3/7 24 h after irradiation. Reprinted with permission from [23], © 2019 WILEY-VCH Verlag GmbH & Co. KGaA, Weinheim, Germany. (**b**) γ-H2AX foci (green) in HUVEC nuclei (stained with DAPI, blue) after 20 Gy irradiation with ^137^Cs source. White bars: 5 µm. Reprinted from [30], with permission from Elsevier. (**c**) γ-H2AX foci overtime referred to figures in b the “−6 h” measure provides a reference before irradiation. The figure also includes data regarding transcriptional inhibition. Reprinted from [30], with permission from Elsevier. (**d**) Example of the tube formation assay in Matrigel^®^ with dermal HMEC. Reprinted with permission from [78]. © 2008 Institut Gustave Roussy UPRES EA 27–10 Journal compilation © 2008 Blackwell Publishing Ltd. (Hoboken, NJ, USA). (**e**) Schematic illustration of the Transwell and the wound healing test for HUVEC migration assessment, in case of low (top) and high (bottom) migration. Reprinted from [57], under the Creative Commons Attribution 4.0 International License. (**f**) Wound healing assay with HUVECs treated with conditioned media from MCF-7, 48 h after the insert removal. Reprinted from [57], under the Creative Commons Attribution 4. 0 International License. (**g**) Images of junction proteins expression in a HUVEC. Left: ZO-1. Right: PECAM. Reprinted from [15], with permission from Elsevier. (**h**) Schematic drawing of the approach found in [106]. Reprinted with permission from [106]. © FASEB. (**i**) Microscope image of the ECs in the network. Scale bar: 250 µm. Figure adapted from [106]. Reprinted with permission from [106]. © FASEB. (**j**) SA-β-Gal activity assay after 6 Gy photon irradiation and its quantification. Reprinted from [107], with permission from Elsevier.

Cell death, proliferation and clonogenic ability are affected by irradiation depending on the dose delivered and the characteristics of the beam. Irradiation-related apoptosis was the most studied effect (43/147 studies, 29.3%), mainly measured through immunofluorescence staining (Figure 4a) or flow cytometry [13,16,23,30,32,46,61,63,66,68,72,73,74,75,76,77,78,79,80,81,82,83,84,85,86,87,88,89,90,91,92,93,94,95,96,97,98,99,100,101,102,103,104,105]. The causal link between IR and apoptosis in ECs has been investigated for years, with examples going back to 25 years ago [103]. The studies highlighted a non-negligible induction of apoptosis depending on the irradiation conditions, including single direct irradiation [13,79,105] or bystander effect [63,65]. Some studies also evaluated the effects of substances for increasing IR-associated apoptosis [80,85] or to decrease it [68,90,93]. In particular, drug treatments, such as statins, which can be administered to treat co-morbidities, caused a decrease in EC apoptosis after irradiation [68,93].

Cell survival can also be measured rather than cell viability, mainly by the colony assay (used in 40 studies, 27.2%) [14,45,58,59,63,70,72,74,76,78,80,85,90,91,92,95,99,102,104,108,109,110,111,112,113,114,115,116,117,118,119,120,121,122,123,124,125,126,127,128]. Clonogenic assays, originally used for cancer cells, represent the gold-standard to measure the biological damage due to IR, under the assumption that the main target for IR is the DNA and cells lethally damaged in their DNA lose their ability to form colonies.

Other possible cell response assays included the MTT assay which measures the metabolic activity of cells (used in 18 studies, 12.2%) [16,19,27,33,67,76,77,88,91,98,123,129,130,131,132,133,134,135], cell counting (eight studies), different proliferation tests (six), live and dead assay (four), and Alamar blue assay (three studies). Cell proliferation was lower in irradiated ECs, even if the rate of reduction was heterogeneous in the studies, also depending on the dose-delivered and culture time post-IR [27,132,135]. In addition, differences were reported when considering single and multiple irradiations, with a continuous decrease of cell number after IR in the first case and a decrease-regrowth in the second one [67].

The need for growth factors to foster cell growth, especially in more advanced models, may affect cell survival after IR, namely having a protective or sensitizing effect. For instance, fibroblast growth factor (bFGF) was found to enhance the survival of irradiated HUVECs [100]. On the other hand, the effect of vascular endothelial growth factor (VEGF) is still not clear. Some studies have suggested a protective effect (see, for instance [124]). Conversely, the protection has been questioned in other works [104].

### 4.2. DNA Damage

DNA damage is the main endpoint of IR. Different theories and models are available to describe this phenomenon, starting from phenomenological to mechanistic ones. We refer interested readers to [136]. In-vitro, DNA damage can be analyzed by using different methods. The most common (17 studies, 11.6%) was the characterization of DNA double-strand breaks (DSB) [23,30,58,68,73,79,89,90,102,112,116,128,132,135,137,138,139]. Such an analysis was usually performed through fluorescence staining after sample fixation (Figure 4b,c). Anyway, a comparison between the analysis of DSB by γ-H2AX foci by using a fluorescence microscope or flow cytometry showed a good correlation between the two techniques [138]. The time-evolution of DSBs in the cells can be evaluated to study cell repair capability [23]. Furthermore, in vitro models can be used to test the ability of compounds to protect DNA from DSB after IR [68]. On the other hand, we must consider that the use of γ-H2AX foci might be misleading when studying radioprotective compounds, whose final aim is to prevent cell death. Indeed, γ-H2AX foci can result from processes outside the formation and repair of radiation-induced DSBs, and their presence can be not correlated with cell survival [128].

Among the other methods available for quantification of DNA damage, the Comet assay was the most commonly used (six studies, 4.1%) [14,30,102,126,127,140]. Such an electrophoresis-based technique measures the DNA strand breaks, but it requires retrieving the cells from their original culture site, which can be difficult in advanced models, with cells embedded in a gel.

### 4.3. Angiogenesis

Differences in angiogenesis pre-/post-irradiation can be evaluated. The most common technique was the so-called tube formation assay (34 studies, 23.1%) [19,33,34,35,37,39,42,53,58,59,76,78,83,84,85,91,92,94,96,108,109,110,115,120,122,123,126,132,141,142,143,144,145]. Briefly, ECs are embedded or seeded in a gel and observed in the subsequent days while organizing a network in a vasculogenesis-like approach (Figure 4d). Such tests, usually performed with Matrigel^®^, result in a non-perfusable network but allow to determine the cell’s ability to form microvascular networks in vitro. In particular, these tests were used to study the inhibition of angiogenesis by compounds administered with irradiation [78,83,96,108], and also ME influence when performing irradiation [58,59]. The relation between angiogenic potential and IR dose rate was investigated, showing that the network formation depended on the dose rate, at least for the low dose rates included in the study [53]. Another option to study angiogenic potential was represented by angiogenic sprouting assays [113]. In this test, an ECs monolayer is seeded on the side of a gel, stimulated with different factors (e.g., VEGF), inducing the migration of ECs in the gel, which originate sprouting that can then be quantified. Angiogenic sprouting was reduced by irradiation, as shown considering a single dose up to 5 Gy [113].

### 4.4. Migration

EC migration is analyzed in vitro to evaluate interactions with other cells or stimuli and study possible pathways involved in angiogenesis during tissue revascularizations (e.g., wound healing). The most common method to study this phenomenon involved migration assays (23 studies, 15.6%) [33,34,35,37,39,57,58,62,64,76,84,85,94,97,107,109,110,119,123,146,147,148,149]. Briefly, in such assay, cells were seeded on a porous membrane, and a chemotactic stimulus is then added on the other membrane side (Figure 4e). After a determined time, cell migration was evaluated by counting the number or the portion of the cells crossing the membrane.

The so-called wound healing assay represented a second possibility for migration tests (nine studies, 6.1%) [39,57,58,107,110,123,132,135,149]. In this case, a wound (i.e., a gap) was created in a cell monolayer and monitored over time to evaluate the cells’ ability to migrate, filling the gap (Figure 4e,f). Alteration of EC migration after irradiation was evaluated in vitro, considering different kinds of stimuli. For instance, an enhanced migration was found when ECs were exposed to tumor-secreted growth factors, actually highlighting possible interaction occurring in the ME [97]. Additionally, the inhibitory action of some compounds on EC migration was evaluated in different studies [33,84,85,109,119,121,148].

### 4.5. EC Barrier

The ability of ECs to act as a semi-permeable membrane is one of the most important characteristics to be investigated when evaluating IR damages to the microvasculature. Indeed, alterations of the EC barrier may lead to an inadequate supply to the tissue. Even if such alterations can be evaluated in vitro through different approaches, they were rarely analyzed.

In these few cases, it was mainly evaluated through the Transwell assay (three studies, 4.8%) [36,38,42]. In this assay, ECs were grown until confluence on a membrane, forming a barrier between the compartments. A compound was loaded in the top compartment, and its concentration was monitored in the bottom one over time. The most common choice for the test compound was fluorescent-labeled dextran, being available at different molecular weights and measurable by fluorescent intensity. After irradiation, the monolayer’s permeability increased in all the three studies analyzed, irrespectively from the EC source, indicating a similar reaction of lymphatic and blood barriers to IR. In all three cases, the increased permeability was associated with loosened cellular junctions and cytoskeletal alterations, evaluated with analysis of specific markers (Figure 4g). In particular, F-actin anisotropy, β-catenin decrease, VE-cadherin degradation and internalization, and loss of PECAM-1 were shown after IR application on the endothelial barrier. Few other markers for cellular junctions were analyzed in other studies, such as zonula-occludens-1 (ZO-1) and claudin-5 [25]. Overall, these studies suggested a detrimental effect of IR on junction proteins, which eventually generated an increase in permeability. Noteworthy, IR effects on ECs junction disruption were highly dependent on the in vitro model’s geometry analyzed [23], underlining the importance of the choice of 2D vs. 3D models for its evaluation. As an example, a particularly relevant approach to measure 3D permeability was proposed, based on fabricated microvascular networks embedded in a microfluidic device, derived from in-vivo data [106] (Figure 4h,i). Through this novel method, the increase in EC permeability after irradiation was reverted by PKCδ inhibition by downregulating IR-induced P-selectin, ICAM 1, and VCAM-1. Since impaired barrier function might also contribute to increased cell extravasation, the authors also quantified neutrophil extravasation, which decreased after PKCδ inhibition. A similar increase of cell extravasation through ECs monolayer after irradiation was also found for cancer cells. Such an increase has important implications for tumor spreading since extravasation is a critical step in metastasis formation [38].

In a couple of studies, the change in the barrier function after irradiation was assessed with trans-endothelial electric resistance (TEER) [15,42], whereby the electric resistance of a monolayer of cells was measured and used as a descriptor of its permeability. Interestingly, changes in TEER presented very different dynamics following different types of irradiation. In particular, photon irradiation caused a sharp drop in TEER, i.e., a decrease in the barrier function after 3 h. In contrast, the application of helium-ion and proton irradiation did not induce these effects [42].

### 4.6. Inflammation

Inflammation was mainly addressed by quantifying cytokines released in culture medium through ELISA assays or specific kits (e.g., intracellular ROS kit). The most commonly studied cytokines were interleukins (IL), analyzed in 20 studies (13.6%) [21,23,29,32,46,48,49,50,53,69,99,126,145,146,147,150,151,152,153,154]. IR application to ECs increased production of specific cytokines such as IL-1α, mediating bystander effects [99], and upregulated pro-inflammatory cytokines and chemokines (including IL-6, IL-1α, and MCP-1), indicators of cell senescence [126]. Interestingly, IL production by ECs increased non-linearly with increasing doses, as shown for IL-8 [151]. This response could be due to other cellular components that might affect IL production, such as ASC producing IL-6 [150]. The presence of other cytokines may also influence IL production, as seen in [29], in which IL-4 and IL-10 affected IL-6 and IL-8 production due to radiation. Possible biological pathways involved in IL production after irradiation have been proposed, including p38/MAPK and NF-κB [50] or thrombomodulin [49,50,145,146], also evaluating their possible inhibition through different agents ([49,145,146,154]). p38 pathway was also involved in ECs bystander effect: irradiated macrophages increased NO production, activating the p38 pathway in ECs, leading to increased apoptosis and inflammatory response [63].

Another important sign of inflammation was the presence of reactive oxygen species (ROS). Such molecules could be found and evaluated intra- and extra-cellularly and cause cellular damage [155]. A first kind of analysis conducted was aimed at identifying the action of antioxidants in ROS reduction [75,135], or as radioprotective agents [14,111]. Among different biological pathways involved, identified using in vitro models [98,105,126,131], a particular example concerned the bystander effect, which can be mediated by extracellular DNA oxidation [65]. Overall, these studies supported the activation of an inflammatory response in ECs after irradiation, mediated by different cytokines, either due to direct irradiation or involving the bystander effect.

### 4.7. Gene Expression and Senescence

PCR-based analysis to evaluate gene expression was considered in 36 different studies (24.5%) [16,17,20,25,26,27,33,37,38,46,50,62,65,68,84,89,107,118,120,122,126,132,134,139,142,144,148,149,151,156,157,158,159,160,161,162] with a few studies reporting extensive gene expression analyses. Bravatà and colleagues evaluated gene expression of different cell lines, both cancer and healthy cells, concluding that gene expression changes were cell line dependent [158]. In another study, the up-regulation of E- and P-selectin, ICAM-1, PECAM-1, and VCAM-1 gene expression in ECs was found to be dose-dependent [16]. In a more extensive study, expression changed in 111 genes [159], with over-expression of genes involved in coagulation and peroxidase activity and structural constituent of ribosomes. Conversely, genes related to regulatory kinase activities were downregulated by irradiation.

Two different studies pointed out similarities in gene expression profiles between irradiated and senescent ECs [132,160]. Such similarities are reflected in functional analogies such as the impairment of angiogenic capacity, an increase of DNA damage, and a decrease of DNA repair capability [126]. In addition, as seen, for instance, with the β-Galactosidase Activity Assay, factors secreted by ECs due to irradiation were similar to those produced by senescent ECs, possibly affecting the ME [123] and contributing to cancer cell aggressiveness [107] (Figure 4j). We refer the reader interested in the mechanism involved in IR-related senesce to [163].

## 5. Brief Discussion and Suggestions

In this review, we analyzed the information available in the literature regarding in-vitro models to evaluate microvascular damage due to ionizing radiation. In-vitro modeling was growing in the last decade, also considering the recommended shift towards non-animal approaches in science. In this context, the 3Rs paradigm has been defined, aiming at Replacement, Refinement, and Reduction of animal experiments [164]. More precisely, the in-vitro model generally provides the possibility to replace animals. However, this was not always the case in the dataset of the papers considered in this review. In 46 studies (31.3%), the in-vitro models were used to support animal studies and prompt further investigation of a particular phenomenon involved in the experiments.

In vitro-models mimic the (3D) structure of a tissue or organ (microvasculature, for this review’s interest) and represent the physiological conditions and the structural microenvironment, providing more predictive in vitro assays compared to cell cultures. They allow the control of testing conditions, often with precise control. Even the effect of different agents (compounds, drugs, cells) can be easily studied, adding and removing them. Their main limitation is their inability to recapitulate the entire physiological system. Therefore, they usually fail in predicting the systemic response to treatment. Further, the heterogeneity of subjects found in the clinical studies is usually not included in in-vitro modeling.

The review addressed the three main steps required to perform this kind of experiments:

(i) production of the in vitro model,
(ii) irradiation, and
(iii) analysis of the samples.

The production step includes classical and advanced models. Even though the use of advanced models (e.g., inclusion of other cell types and use of 3D techniques) aims to describe the ME better, it results in more complex operations and increased technologies required to build the model [6]. As an example, a 2D culture in a flask is far less complex than a network-on-a-chip approach [11,23]. Additionally, classical and advanced models often differ for the time required to prepare them. For instance, overnight culturing may be sufficient to create an ECs monolayer in a well. In contrast, networks on a chip can take some days (usually < 7) to be ready for treatments and analyses. Although advanced in-vitro models are more challenging to handle, they usually better recapitulate features characterizing ME from a 3D environment to the heterogeneous cell population. Such features are not of secondary importance, given that the result of the analyses might be affected by their presence [22,23]. Consequently, we suggest advanced models for future experiments, recapitulating as many ME characteristics as possible (e.g., 3D ME, cellular type other than ECs). In particular, we emphasize the critical role of advanced microfluidic models in this scenario, providing a suitable platform to recapitulate the ME complexity.

Considering the irradiation step, we noticed that some important information or appropriate irradiation setting are often disregarded (see Figure 2). This might lead to a difficult interpretation of results or, in the worst scenario, inaccuracies in the assessment of the delivered dose. We strongly suggest that future experiments consider and report all the necessary information about the irradiation process, including the dose delivered, the dose rate, the energy of the photon beam, the method of irradiation, the possible fractionation, and the strategy to avoid the build-up region (e.g., phantoms).

Lastly, the analysis step aims at investigating the different phenomena involved (see Figure 3 and Figure 4). We grouped the possible analyses by the endpoint evaluated. For each of them, the main techniques have been reported. However, we recall that different methods may be available based on the type of in-vitro model considered. For instance, colony assay can be easily used with 2D models, but not with 3D gel embedded ones. Methods for the analysis should be appropriately chosen for the considered in vitro model and the study outcome.

As a final remark, the investigation problem considered in this review is characterized by a strong inter-disciplinarity, requiring knowledge on in-vitro models production and analysis (biological field, engineering) and radiobiological analyses and irradiation protocols (medical physics). We strongly suggest researchers cover all the required skills when considering the composition of the research team.

## 6. Materials and Methods

The systematic searches were conducted by using PUBMED [165] and Elsevier Scopus [166] on 29/12/2020. L.P. designed the search strategy with the following strings. Pubmed: “((microvascular cell*) OR haec* OR huvec* OR HMEC* OR HDMEC*) AND (radiotherapy OR (ionizing radiation) OR (radiation toxicity) OR (radiation damage)) AND (culture OR co-culture OR microfluidic OR on-chip) NOT (UV OR ultra-violet OR chemotherap*)”; Scopus: “TITLE-ABS-KEY ((microvascular AND cell*) OR haec* OR huvec* OR hmec* OR hdmec*) AND TITLE-ABS-KEY (radiotherapy OR (ionizing AND radiation) OR (radiation toxicity) OR (radiation damage)) AND TITLE-ABS-KEY (culture OR co-culture OR microfluidic OR on-chip) AND (LIMIT-TO (DOCTYPE, “ar”)) ANDNOT TITLE-ABS-KEY (UV OR ultra-violet OR chemotherap*)”. There was no limit applied by publication year.

The two searches resulted in 235 and 162 papers, respectively, for a total of 397 references. After the first step of duplicates removal, i.e., articles found in both the search engines, the total number of references was 276. Then, two different selections were based on the title/abstract and on the full text, respectively. We defined the following criteria: (i) presence of in vitro test; (ii) presence of endothelial cells; (iii) presence of the photon irradiation; (iv) availability of English full text. The total number of references was 241 after the selection based on title/abstract and 147 after the full text-based selection.

These 147 papers were randomly divided into 4 groups. Each group was read by one of the following authors [L.P., L.M., A.R., and V.S.]. Discussions were conducted prior, during, and after the reading to unify the classification. All the metrics shown in the review have been computed by a spreadsheet in Microsoft Excel. Graphs were made by using Microsoft Excel and GraphPad Prism 9 (GraphPad Software, San Diego, CA, USA).

## 7. Conclusions

In this work, we systematically reviewed the literature about in-vitro models to assess microvascular damage due to IR. In particular, we analyzed how they are produced, irradiated, and analyzed. The analysis has shown different possibilities across the three steps, highlighting the most common techniques and new emerging methods.

We showed the importance of recapitulating important ME features in the production phase, such as 3D environment, heterogeneous cell population, and chemical composition (e.g., growth factors). These features are essential to model IR damage to EC accurately. In this direction, we stress the possibilities enabled by advanced in vitro models and microfluidic systems.

Besides, we identified some critical information and peculiar needs for the irradiation part, which are often not considered in the experiments or reported in the manuscripts. We recommend to future authors to include the dose delivered, the dose rate, the beam’s energy, and the techniques to handle the build-up region. Additionally, the scheme and the irradiation’s modality (direct irradiation and fractionation) must be defined.

Lastly, different analysis techniques are here reported and grouped for their outcome. These represent the state-of-the-art, and they should be considered depending on the specific model used.

## Data Availability

No new data were created in this study. Data sharing is not applicable to this article.
